# Machine Learning–Derived Acetabular Dysplasia and Cam Morphology Are Features of Severe Hip Osteoarthritis: Findings From UK Biobank

**DOI:** 10.1002/jbmr.4649

**Published:** 2022-08-07

**Authors:** Monika Frysz, Benjamin G Faber, Raja Ebsim, Fiona R Saunders, Claudia Lindner, Jennifer S Gregory, Richard M Aspden, Nicholas C Harvey, Tim Cootes, Jon H Tobias

**Affiliations:** ^1^ Musculoskeletal Research Unit University of Bristol Bristol UK; ^2^ Medical Research Council Integrative Epidemiology Unit University of Bristol Bristol UK; ^3^ Division of Informatics, Imaging and Data Sciences The University of Manchester Manchester UK; ^4^ Centre for Arthritis and Musculoskeletal Health University of Aberdeen Aberdeen UK; ^5^ Medical Research Council Lifecourse Epidemiology Centre University of Southampton Southampton UK; ^6^ NIHR Southampton Biomedical Research Centre University of Southampton and University Hospitals Southampton NHS Foundation Trust Southampton UK

**Keywords:** OSTEOARTHRITIS, HIP SHAPE, STATISTICAL SHAPE MODELING, CAM MORPHOLOGY, ACETABULAR DYSPLASIA

## Abstract

The contribution of shape changes to hip osteoarthritis (HOA) remains unclear, as is the extent to which these vary according to HOA severity. In the present study, we used statistical shape modeling (SSM) to evaluate relationships between hip shape and HOA of different severities using UK Biobank DXA images. We performed a cross‐sectional study in individuals with left hip dual‐energy X‐ray absorptiometry (DXA) scans. Statistical shape modeling (SSM) was used to quantify hip shape. Radiographic HOA (rHOA) was classified using osteophyte size and number and joint space narrowing. HOA outcomes ranged in severity from moderate (grade 2) to severe (grade ≥3) rHOA, hospital‐diagnosed HOA, and subsequent total hip replacement (THR). Confounder‐adjusted logistic regression between the top 10 hip shape modes (HSMs) and OA outcomes was performed. Further models adjusted for alpha angle (AA) and lateral center‐edge angle (LCEA), reflecting acetabular dysplasia and cam morphology, respectively. Composite HSM figures were produced combining HSMs associated with separate OA outcomes. A total of 40,311 individuals were included (mean 63.7 years, 47.8% male), of whom 5.7% had grade 2 rHOA, 1.7% grade ≥3 rHOA, 1.3% hospital‐diagnosed HOA, and 0.6% underwent THR. Composite HSM figures for grade 2 rHOA revealed femoral neck widening, increased acetabular coverage, and enlarged lesser and greater trochanters. In contrast, grade ≥3 rHOA, hospital‐diagnosed HOA, and THR were suggestive of cam morphology and reduced acetabular coverage. Associations between HSMs depicting cam morphology and reduced acetabular coverage and more severe HOA were attenuated by AA and LCEA adjustment, respectively. Relationships between hip shape and HOA differed according to severity. Notably, cam morphology and acetabular dysplasia were features of severe HOA, but unrelated to moderate disease, suggesting possible prognostic utility. © 2022 The Authors. *Journal of Bone and Mineral Research* published by Wiley Periodicals LLC on behalf of American Society for Bone and Mineral Research (ASBMR).

## Introduction

Hip osteoarthritis (HOA) is a common age‐related condition in which progressive changes develop in the cartilage and surrounding bone, leading to pain and stiffness, loss of function, and ultimately total hip replacement (THR).^(^ ^1^ ^)^ Abnormalities in hip shape are thought to represent an important predisposing factor for the development of HOA, as illustrated by developmental dysplasia of the hip (DDH) which is linked to early onset HOA.^(^ ^2^, ^3^ ^)^ There has also been considerable interest in the contribution of more subtle changes in hip shape to the development of HOA, which occur more commonly. These include cam and pincer morphologies, which are thought to lead to femoro‐acetabular impingement (FAI), and acetabular dysplasia.^(^ ^4^, ^5^ ^)^

The above characteristics are frequently defined by geometric measurements on pelvic radiographs, such as alpha angle (AA) used to define cam morphology, and lateral centre‐edge angle (LCEA) to define pincer morphology/acetabular dysplasia. Radiographic shape changes have also been examined in relation to HOA using statistical shape modeling (SSM) to characterize global hip shape.^(^ ^6^ ^)^ SSM has identified relationships between HOA and a range of shape changes besides cam and pincer‐type morphology, including femoral head size, femoral neck (FN) width, and lesser and greater trochanter size.^(^ ^4^, ^7^ ^)^ A possible explanation for the heterogeneous findings from different studies is the varying definitions used for HOA, including radiographic HOA (rHOA), symptomatic rHOA, and end‐stage disease as reflected by THR.

Characterizing how the hip shape‐HOA relationship varies between hip shape and HOA according to HOA severity has important implications for understanding pathogenesis and developing interventions. For example, surgery to remove cam‐type lesions has been advocated to treat FAI and reduce progression of HOA.^(^ ^8^, ^9^ ^)^ However, cam‐type morphology in severe HOA might reflect a consequence rather than a cause of end‐stage HOA, undermining the rationale for their removal. We are not aware of any previous studies where cam‐type hip shape changes have been followed longitudinally as HOA progresses, to examine the temporality of these relationships. However, a cross‐sectional study in individuals with HOA of different severities permits a degree of inference.

Having developed a novel semi‐automated machine learning method to quantify hip shape in over 40,000 hip dual‐energy X‐ray absorptiometry (DXA) scans in UK Biobank, we aimed to evaluate how SSM‐derived hip shape relates to HOA of different levels of severity. To understand the contribution of cam and pincer morphologies/acetabular dysplasia to associations which we observed, we also aimed to determine to what extent hip shape‐HOA relationships are modified by adjustment for AA or LCEA. Improved understanding of the relationship between hip shape and HOA could then provide a basis for the development of new interventions to reduce HOA progression by targeting specific aspects of hip morphology.

## Subjects and Methods

### Study population

The UK Biobank (UKB) is a prospective cohort study with phenotypic and genetic data collected on approximately 500,000 individuals from the United Kingdom, aged between 40 and 69 years at the time of recruitment (2006–2010).^(^ ^10^ ^)^ The UKB Ethics Advisory Committee oversees the maintenance, development, and use of UKB data and its approval covers this study. All subjects provided informed consent before participation. As part of the Imaging Enhancement study^(^ ^11^ ^)^ (follow‐up 2), which began in 2014, high‐resolution iDXA scans (iDXA GE‐Lunar, Madison, WI, USA) (both hips, knees, lateral and lumbar spine, and total body) are being collected. As of April 2021, DXA images are available for 42,441 participants of which 41,160 were left hip images.

### Statistical shape model of the proximal femur

An initial training sample of 2000 and a further extension sample of 5000 individuals with a left hip DXA image were selected in January 2019 to train an automated model for point placement (search model). Both samples were enriched for self‐reported OA to increase the number of diverse and pathological scans as part of a wider research program, which aims to automate the assessment of radiographic OA. This was achieved by ensuring 20% of the training and extension samples consisted of individuals with self‐reported OA. The remaining sample comprised randomly selected individuals, equally split between males and females. Of the 2000 images from the initial training sample, 70 were excluded, and of the 5000 images in the extension sample, 167 were excluded. Exclusions were attributable to poor image quality, image error, or withdrawal of consent.

**Fig. 1:**
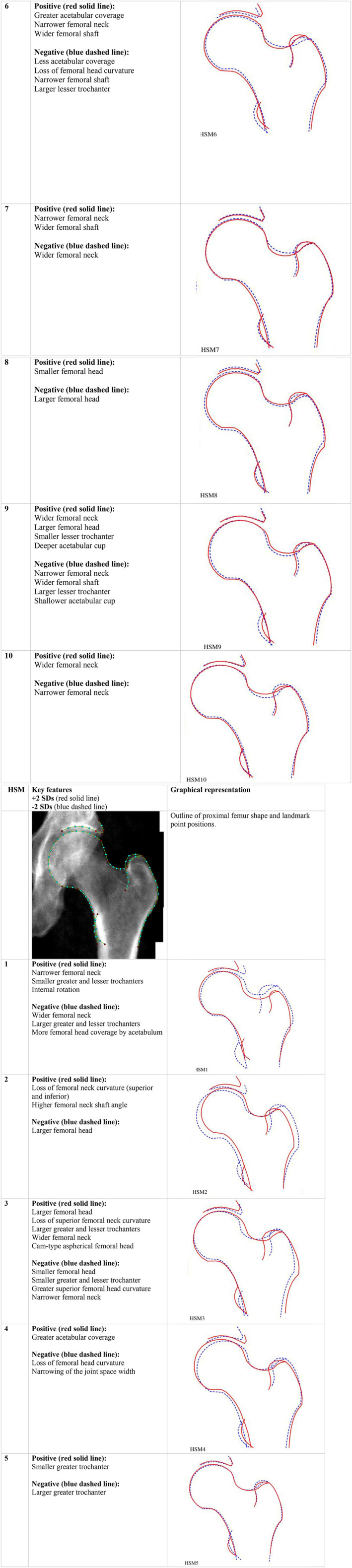
Outline of proximal femur shape and landmark point positions (key landmark points are shown in red) and variation described by the top 10 hip shape modes (HSMs).

Eighty‐five landmark points (including 19 key points) were defined to outline the femoral head, metaphysis, lesser and greater trochanters, and the superior acetabulum (Fig. 1). The outline did not encompass osteophytes. Images from the initial training sample were marked up with these points by trained annotators. An automated random forest‐based machine‐learning search model was then trained to place points on new unseen images.^(^ ^12^, ^13^ ^)^ Images from the extension sample were subsequently annotated by the trained search model. After point placement, each image was visually assessed and point placement adjusted if necessary.

A final search model trained on 6763 images (further 44 images with points placed on the edge of an image were excluded from the search model but included in final analysis) was subsequently used to annotate all available and not already annotated left hip DXA images. Each image was then visually inspected by one trained marker (BGF, rheumatology doctor), and points were adjusted if necessary; in cases of uncertainty or where an osteophyte was present, a second reader (FS, postdoctoral researcher) was consulted. Osteophytes were marked up at the lateral acetabulum, superolateral femoral head, and inferomedial femoral head using a custom tool (The University of Manchester). Where an osteophyte was present, the landmark points were placed inside of the osteophyte margin (as previously described^(^ ^14^ ^)^). FS reviewed 2857 (8.5%) images, of which 214 (0.6%) were discussed between FS and BGF, resulting in 69 (0.2%) changes. Of 33,533 images with automatically placed points, only 3417 (10.2%) images required any correction, and where a point was moved, the average distance was 1.94 mm.

After point placement, an SSM was built from all available images, producing a set of orthogonal modes of variation known as principal components (hip shape modes [HSMs]).^(^ ^15^, ^16^ ^)^ Together, all modes explain 100% of variance in the data set, with the first HSM accounting for the largest amount of variance and subsequent HSMs accounting for less variance. Each HSM is standardized to a mean = 0 and standard deviation (SD) = 1. For statistical analysis, the first 10 HSMs were selected, which together explained 86.3% of hip shape variance.

### OA outcomes

Hospital‐diagnosed HOA and THR (affected hip not specified) were obtained from hospital episode statistics (HES) data. All clinical data in HES are coded according to the World Health Organization's International Classification of Disease (ICD) codes. All operations and procedures are coded according to the Office of Population, Censuses and Surveys: Classification of Interventions and Procedures (OPCS). HES data linked to the UKB resource covers periods before and after the DXA assessment. As previously described, osteophytes at the lateral acetabulum, superior‐lateral, and inferior‐medial femoral head were manually shaded in a custom‐made tool (University of Manchester).^(^ ^14^ ^)^ Based on area (in mm^2^) and subsequent thresholds, each osteophyte was graded from 0–3. Minimum joint space width (mJSW) was automatically calculated (in mm) using a custom Python script applied to the template points placed around the superior femoral head (points 22–31) and acetabulum (points 78–84). Subsequent thresholds of height‐adjusted mJSW were categorized into JSN grades 0–3. We have since derived four grades of overall rHOA from the sum of osteophyte and mJSW grades (total 12).^(^ ^17^ ^)^ In the present study, we used moderate rHOA (grade 2 only) and rHOA grade ≥3, as outcomes.

### Covariates

Both AA and LCEA were derived automatically from hip DXA images (AP view) using Python.^(^ ^18^ ^)^ To derive AA, a circle was first fitted using femoral head points 15–28 (Fig. 1). The angle was then measured between a line running through the center of the femoral head and neck and a line passing through the center of the femoral head and the point at which the femoral head–neck junction leaves the circle. The Python script to automatically derive AA is openly available.^(^ ^19^, ^20^ ^)^ LCEA was calculated from a line passing through the lateral edge of the acetabulum (outline point 78; Fig. 1) and the center of the femoral head, and a line that passes perpendicular to the image *x*‐axis through the center of the femoral head. For more details for derivation of AA and LCEA, see Faber and colleagues.^(^ ^18^ ^)^ We considered age, sex, height, weight, and ethnicity as potential confounders. Height and weight were measured at a time of DXA imaging, and ethnic background was ascertained from a touchscreen questionnaire completed during the assessment center visit.

### Statistical analysis

Descriptive statistics are shown as mean (SD) and counts with percentages (%) for continuous and categorical variables, respectively. Associations with OA outcomes were analyzed using logistic regression (hospital‐diagnosed HOA, moderate and grade ≥3 rHOA) and Cox proportional hazards regression (THR). Unadjusted and confounder‐adjusted (age, sex, height, weight, and ethnicity) results are shown as odds ratios (ORs)/hazard ratios (HRs) per 1 SD change in exposure, with 95% confidence intervals (CIs). Further adjustment was made for AA and LCEA. We applied Bonferroni corrected *p* value threshold of 0.005 (0.05/10 HSMs tested). All statistical analyses were performed in Stata 16.0. To illustrate the overall effect of hip shape on each OA outcome, composite HSM figures were plotted by combining all HSMs associated with each outcome at *p* value threshold of <0.005. Briefly, confounder‐adjusted beta coefficients (Model 2) (instead of odds ratios [ORs]) for HSM‐OA associations were generated. Each beta was multiplied by the non‐standardized HSM‐specific SD to account for the contribution of the HSM to overall variance in shape. An arbitrary multiplication factor of 5 was also applied to enable shapes to be visualized more clearly. These values were subsequently combined into one vector to model the overall effect of hip shape on each OA outcome.

## Results

Of 41,160 available left hip DXA images, 820 were removed because of either poor image quality, image error, or withdrawal of consent. Subsequently, HSM score data were generated for 40,340 individuals. Of those, 40,311 had complete outcome and covariate data, on which further analyses were based, comprising 19,290 (47.9%) males and 21,021 (52.1%) females, mean age of 64 years (SD 7.6) (Table 1). On grading DXA images of study participants for rHOA, 2314 (5.7%) had rHOA grade 2 and 700 (1.7%) rHOA grade ≥3. A total of 527 (1.3%) study participants had hospital‐diagnosed HOA, of whom 127 were diagnosed before the DXA scan and 400 afterwards. In addition, 259 (0.6%) participants subsequently underwent a THR at a mean of 3.2 years after the DXA scan. The first 10 HSMs explained 86.3% of total variance in the data set (see Supplemental Table S1 for individual and cumulative variance). For visual representation (Supplemental Figs. S1 and S2) and variation described by each HSM, refer to Fig. 1.

**Table 1:** Characteristics of UK Biobank Study Participants

		Combined *N* = 40,311	Males *n* = 19,290	Females *n* = 21,021
		Mean (SD)	Mean (SD)	Mean (SD)
Age (years)		63.7 (7.6)	64.3 (7.7)	63.0 (7.4)
Weight (kg)		75.4 (15.1)	83.2 (13.4)	68.2 (12.9)
Height (cm)		170.1 (9.4)	177.2 (6.6)	163.6 (6.4)
NSA (°)		134.2 (5.1)	133.0 (4.7)	135.2 (5.2)
NNW (mm)		31.6 (3.5)	34.5 (2.4)	29.0 (2.0)
AA (°)		47.8 (10.8)	51.9 (13.1)	44.0 (5.8)
LCEA (°)		35.7 (7.0)	35.9 (7.0)	35.5 (7.0)
		*n* (%)	*n* (%)	*n* (%)
rHOA grade 2 only	No	37,997 (94.3)	17,713 (91.8)	20,284 (96.5)
	Yes	2314 (5.7)	1577 (8.2)	737 (3.5)
rHOA grade ≥3	No	39,611 (98.3)	18,781 (97.4)	20,830 (99.1)
	Yes	700 (1.7)	509 (2.6)	191 (0.9)
Hospital‐diagnosed HOA	No	39,784 (98.7)	19,070 (98.9)	20,714 (98.5)
	Yes	527 (1.3)	220 (1.1)	307 (1.5)
THR	No	40,051 (99.4)	19,184 (99.5)	20,868 (99.3)
	Yes	259 (0.6)	106 (0.6)	153 (0.7)
Ethnicity	White	39,020 (96.8)	18,646 (96.7)	20,374 (96.9)
	Asian	437 (1.1)	266 (1.4)	171 (0.8)
	Black	253 (0.6)	119 (0.6)	134 (0.6)
	Mixed	178 (0.4)	61 (0.3)	117 (0.6)
	Chinese	116 (0.3)	51 (0.3)	65 (0.3)

AA = alpha angle; HOA = hip osteoarthritis; HSM = hip shape mode; LCEA = lateral center‐edge angle; NNW = narrowest neck width; NSA = neck shaft angle; rHOA = radiographic hip osteoarthritis; THR = total hip replacement.

### Individual hip shape modes versus OA outcomes

**Fig. 2:**
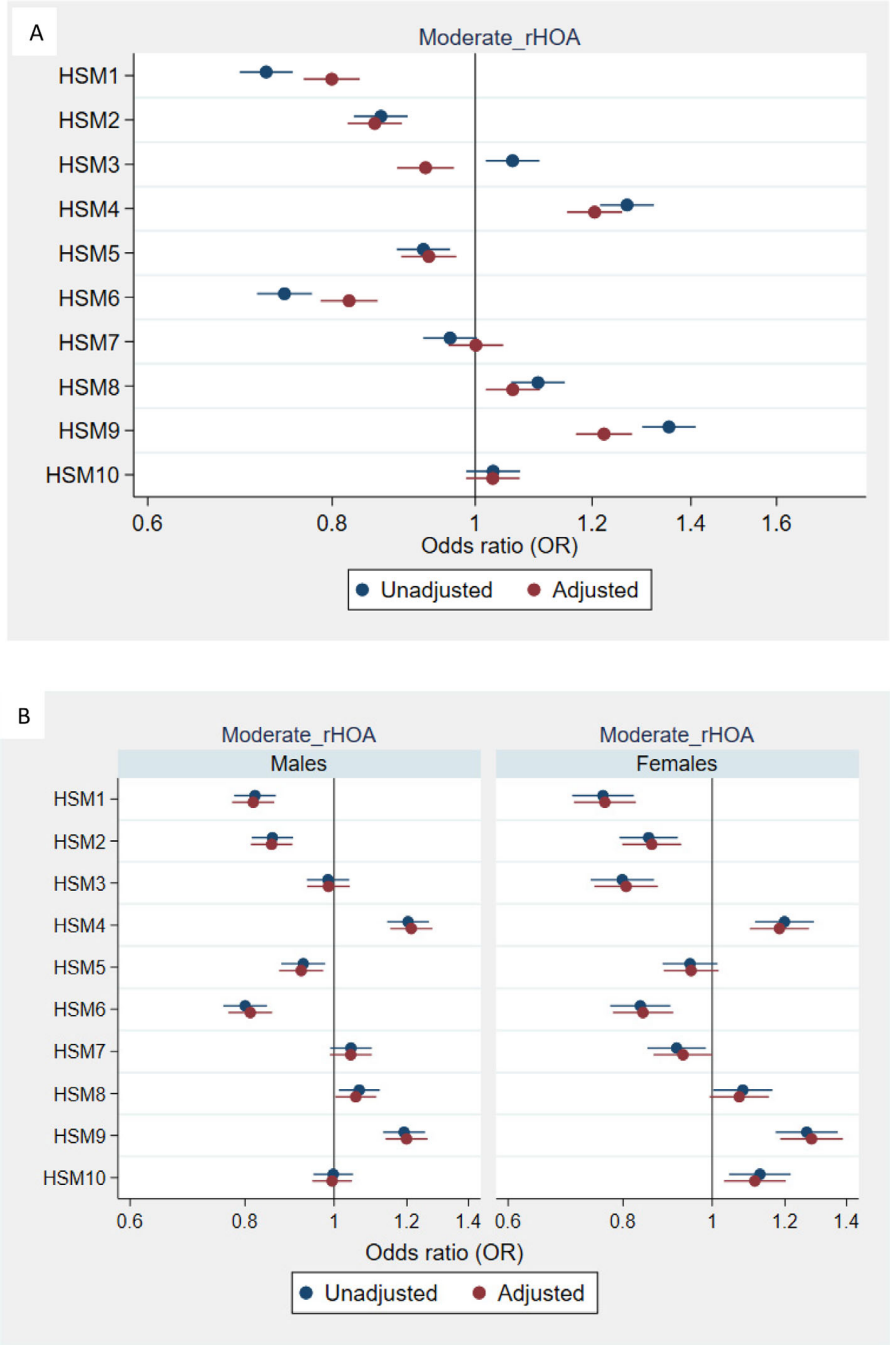
Associations between hip shape and radiographic hip osteoarthritis (HOA) grade 2; combined (*A*) and stratified by sex (*B*). Results are odds ratios (ORs) of outcome per SD increase in hip shape mode (HSM) score, 95% confidence interval (CI), and *p* value (*p*). Model adjusted for age, sex, height, weight, and ethnicity (categorized into binary variable white/other).

**Fig. 3:**
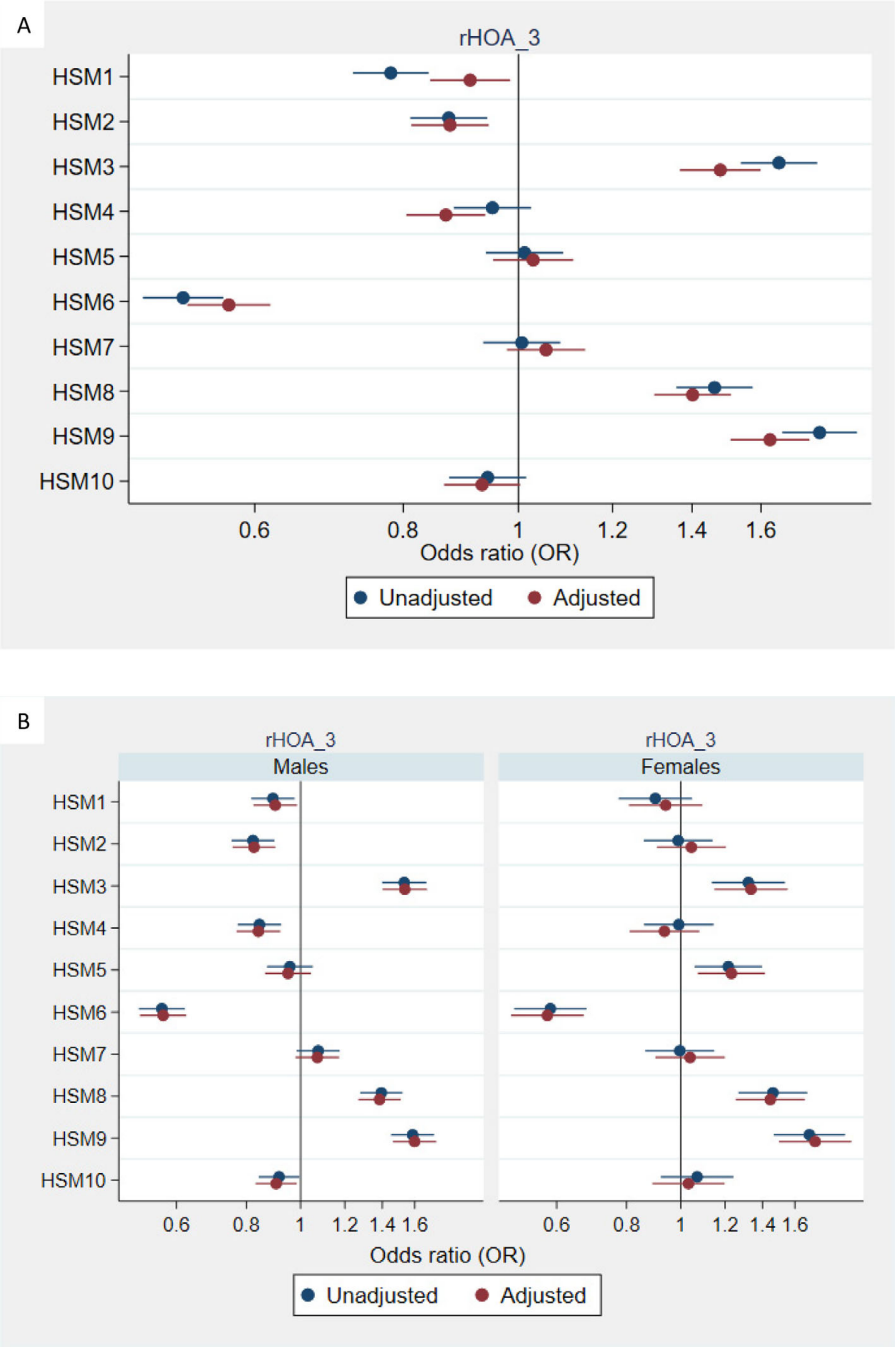
Associations between hip shape and radiographic hip osteoarthritis (HOA) grade ≥3; combined (*A*) and stratified by sex (*B*). Results are odds ratios (ORs) of outcome per SD increase in hip shape mode (HSM) score and 95% confidence interval (CI). Model adjusted for age, sex, height, weight, and ethnicity (categorized into binary variable white/other).

In unadjusted analysis, eight HSMs showed an association with moderate rHOA (grade 2), the majority of which were partially attenuated by adjustment for age, sex, height, weight, and ethnicity (Fig. 2*A*). In sex‐stratified analysis, most results were similar, except associations with HSM3 in females but not in males, and an association between HSM5 and moderate rHOA in males but not in females (Fig. 2*B*). For more severe rHOA (grade ≥3), adjusted sex combined associations with HSM3 and HSM4 were in the opposite direction compared with associations with moderate rHOA (grade 2), whereas associations with HSM8 and HSM9 were considerably stronger (Fig. 3*A*). In sex‐stratified analysis, associations with HSM3, HSM6, HSM8, and HSM9 were consistent in males and females in terms of direction of effect, and there was evidence for an association with HSM2 and HSM4 in males but not females, and association with HSM5 in females but not males (Fig. 3*B*).

**Fig. 4:**
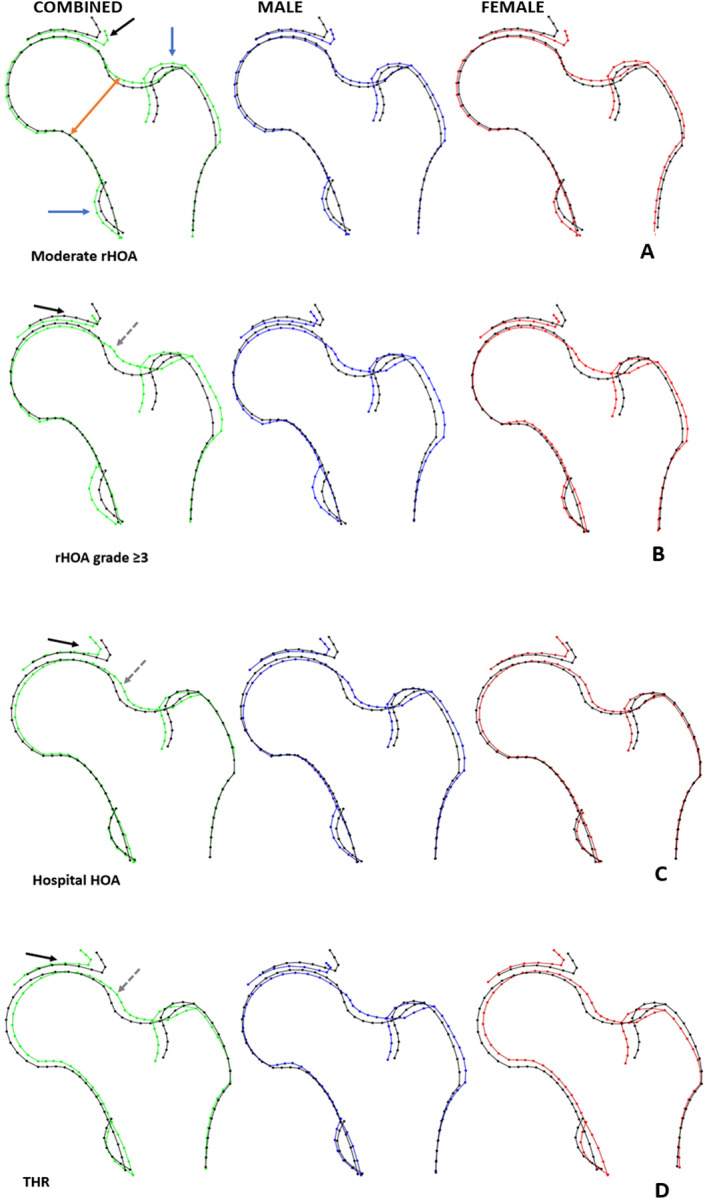
Composite hip shapes illustrating the overall effect of hip shape on osteoarthritis outcomes, generated by combining all HSMs associated with each outcome at *p* value threshold of <0.005. Black outline = mean hip shape; green outline = sex combined OA shape; blue outline = OA shape in males; red outline = OA shape in females. (*A*) Moderate radiographic hip OA (rHOA) (grade 2), (*B*) rHOA grade ≥3, (*C*) hospital‐diagnosed HOA, (*D*) total hip replacement (THR). Arrows indicate regions showing the most pronounced associations with each hip OA outcome: orange arrow = femoral neck width; black arrow = acetabular coverage; blue arrows = lesser and greater trochanter size; gray dashed arrow = changes in the superior head–neck junction suggestive of cam‐type morphology.

Seven HSMs were associated with hospital‐diagnosed HOA, with little difference between unadjusted versus fully adjusted results (Supplemental Fig. S3 *A*). In sex‐stratified results, associations between HSM2 and hospital‐diagnosed HOA were stronger in females, whereas associations between HSM6, HSM7, HSM8, HSM9, and HSM10 and THR were stronger in males (Supplemental Fig. S3 *B*). Hip shape showed similar relationships with THR as those found for hospital‐diagnosed HOA (Fig. 4 *A*, *B*).

### Composite hip shape models and HOA

HSM associations were combined to visualize the overall hip shape associated with each HOA outcome. Hip shape associated with moderate rHOA comprised (i) widening of the femoral neck (FN) due to upward displacement of the superior border, (ii) greater acetabular coverage of the femoral head, (iii) enlargement of the greater trochanter, and (iv) increased size of the lesser trochanter, particularly in males (Fig. 4*A*). The composite shape differed when progressively more severe definitions of OA were utilized (Fig 4*B–D*): (i) the expanded upper surface of the hip was situated more superiorly, involving the lateral border of the femoral head suggestive of cam morphology, (ii) the femoral head, in the superolateral aspect, showed reduced acetabular coverage suggestive of acetabular dysplasia, (iii) in females, the lateral border of the greater trochanter was displaced medially rather than laterally, and the greater trochanter was no longer enlarged, and (iv) lesser trochanter size was no longer increased.

### Additional adjustment for AA

To study to what extent these relationships between hip shape and HOA are explained by cam morphology, we examined the effect of adjustment for AA on our results. AA adjustment slightly attenuated most associations between HSMs and moderate and severe rHOA (grade ≥3) (Table 2). In particular, the increased risk of rHOA grade ≥3 with higher scores of HSM3 was attenuated by more than 60%, in keeping with the fact that cam morphology is a major feature of HSM3 (Supplemental Fig. S1), and the composite shape associated with severe rHOA (grade ≥3) (Fig. 4*B*). In addition, HSM3 is an important risk factor for rHOA grade ≥3 (Fig. 3*A*). Associations between higher scores of HSM3 and increased risk of HOA and THR were attenuated by approximately 30% after AA adjustment (Table 3), consistent with our finding that cam morphology is also a feature of shape associated with hospital‐diagnosed HOA and THR (Fig. 4 *C*, *D*).

**Table 2:** Associations Between Hip Shape and rHOA Adjusted for AA and LCEA

		Model 2	Model 2 + AA	Model 2 + LCEA
HSM	Outcome	OR (95% CI)	OR (95% CI)	OR (95% CI)
1	Moderate rHOA	0.80 (0.76, 0.83)*	0.81 (0.78, 0.85)*	0.89 (0.85, 0.94)*
2	Moderate rHOA	0.86 (0.82, 0.89)*	0.90 (0.86, 0.94)*	0.97 (0.93, 1.01)
3	Moderate rHOA	0.92 (0.88, 0.97)*	0.82 (0.79, 0.86)*	1.06 (1.01, 1.11)
4	Moderate rHOA	1.20 (1.15, 1.26)*	1.19 (1.14, 1.25)*	1.03 (0.98, 1.08)
5	Moderate rHOA	0.93 (0.89, 0.97)*	0.91 (0.87, 0.95)*	0.91 (0.87, 0.95)*
6	Moderate rHOA	0.82 (0.78, 0.86)*	0.90 (0.86, 0.95)*	0.81 (0.77, 0.85)*
7	Moderate rHOA	1.00 (0.96, 1.04)	1.00 (0.96, 1.04)	1.00 (0.96, 1.05)
8	Moderate rHOA	1.06 (1.02, 1.11)	1.02 (0.98, 1.07)	1.05 (1.01, 1.10)
9	Moderate rHOA	1.22 (1.17, 1.28)*	1.15 (1.10, 1.20)*	1.27 (1.21, 1.32)*
10	Moderate rHOA	1.03 (0.99, 1.07)	1.05 (1.01, 1.09)	1.02 (0.98, 1.07)
1	rHOA ≥3	0.91 (0.85, 0.99)	0.96 (0.88, 1.03)	0.91 (0.84, 0.98)
2	rHOA ≥3	0.87 (0.81, 0.94)*	0.97 (0.90, 1.05)	0.86 (0.79, 0.93)*
3	rHOA ≥3	1.49 (1.37, 1.61)*	1.19 (1.10, 1.29)*	1.55 (1.43, 1.69)*
4	rHOA ≥3	0.87 (0.81, 0.94)*	0.86 (0.79, 0.92)*	0.84 (0.77, 0.91)*
5	rHOA ≥3	1.03 (0.95, 1.11)	0.99 (0.92, 1.08)	1.03 (0.95, 1.11)
6	rHOA ≥3	0.57 (0.53, 0.62)*	0.70 (0.64, 0.76)*	0.57 (0.53, 0.62)*
7	rHOA ≥3	1.06 (0.98, 1.14)	1.04 (0.97, 1.12)	1.06 (0.98, 1.14)
8	rHOA ≥3	1.40 (1.30, 1.51)*	1.30 (1.21, 1.41)*	1.40 (1.30, 1.51)*
9	rHOA ≥3	1.63 (1.51, 1.75)*	1.40 (1.30, 1.52)*	1.63 (1.51, 1.76)*
10	rHOA ≥3	0.93 (0.87, 1.00)	0.97 (0.90, 1.05)	0.93 (0.87, 1.00)

AA = alpha angle; LCEA = lateral center‐edge angle; rHOA = radiographic hip OA.

Moderate rHOA: rHOA grade 2 only. Results are odds ratios (ORs) of outcome per SD increase in hip shape mode (HSM) score and 95% confidence interval (CI). Model 2: adjusted for age, sex, height, weight, and ethnicity (categorized into binary variable white/other).

* *p* < 0.005.

**Table 3:** Associations Between Hip Shape and Hospital‐Diagnosed HOA and TJR Adjusted for AA and LCEA

		Model 2	Model 2 + AA	Model 2 + LCEA
HSM	Outcome	OR (95% CI)	OR (95% CI)	OR (95% CI)
1	Hospital HOA	1.00 (0.91, 1.09)	1.02 (0.93, 1.11)	0.91 (0.82, 1.00)
2	Hospital HOA	1.21 (1.11, 1.32)*	1.27 (1.16, 1.39)*	1.13 (1.03, 1.24)
3	Hospital HOA	1.31 (1.20, 1.43)*	1.21 (1.11, 1.33)*	1.23 (1.11, 1.35)*
4	Hospital HOA	0.82 (0.75, 0.89)*	0.81 (0.74, 0.88)*	0.89 (0.81, 0.98)
5	Hospital HOA	1.03 (0.95, 1.12)	1.01 (0.93, 1.10)	1.05 (0.96, 1.14)
6	Hospital HOA	0.81 (0.74, 0.88)*	0.88 (0.80, 0.96)	0.81 (0.74, 0.89)*
7	Hospital HOA	1.18 (1.08, 1.28)*	1.18 (1.08, 1.28)*	1.17 (1.07, 1.28)*
8	Hospital HOA	1.22 (1.12, 1.33)*	1.18 (1.08, 1.28)*	1.23 (1.12, 1.34)*
9	Hospital HOA	1.44 (1.31, 1.57)*	1.36 (1.24, 1.49)*	1.41 (1.29, 1.55)*
10	Hospital HOA	0.93 (0.86, 1.02)	0.95 (0.87, 1.03)	0.94 (0.86, 1.03)
		HR (95% CI)	HR (95% CI)	HR (95% CI)
1	THR	1.07 (0.94, 1.22)	1.09 (0.96, 1.23)	0.92 (0.81, 1.06)
2	THR	1.42 (1.25, 1.60)*	1.50 (1.33, 1.70)*	1.27 (1.11, 1.45)*
3	THR	1.35 (1.19, 1.53)*	1.25 (1.10, 1.42)*	1.19 (1.04, 1.36)
4	THR	0.81 (0.72, 0.92)*	0.81 (0.71, 0.91)*	0.97 (0.85, 1.12)
5	THR	0.99 (0.88, 1.11)	0.97 (0.86, 1.09)	1.02 (0.90, 1.15)
6	THR	0.70 (0.62, 0.80)*	0.77 (0.68, 0.87)*	0.72 (0.63, 0.81)*
7	THR	1.29 (1.14, 1.45)*	1.28 (1.14, 1.44)*	1.27 (1.13, 1.43)*
8	THR	1.45 (1.29, 1.64)*	1.40 (1.24, 1.58)*	1.45 (1.29, 1.64)*
9	THR	1.59 (1.40, 1.79)*	1.50 (1.33, 1.70)*	1.53 (1.36, 1.74)*
10	THR	0.89 (0.79, 1.00)	0.90 (0.80, 1.02)	0.90 (0.80, 1.02)

AA = alpha angle; LCEA = lateral center‐edge angle; hospital HOA = hospital‐diagnosed hip OA; THR = total hip replacement.

Results are odds ratios (ORs)/hazard ratios (HR) of outcome per SD increase in hip shape mode (HSM) score and 95% confidence interval (CI). Model 2: adjusted for age, sex, height, weight, and ethnicity (categorized into binary variable white/other).

* *p* < 0.005.

### Additional adjustment for LCEA

To study the contribution of pincer morphology/acetabular dysplasia to relationships between hip shape and HOA, we examined the effect of adjustment for LCEA. The association between higher HSM4 and greater odds of moderate rHOA was completely attenuated by adjustment for LCEA, whereas the reduced odds with HSM1 and HSM2 were partially and fully attenuated (Table 2). These findings are in keeping with the fact that HSM1, HSM2, and HSM4 depict pincer‐type morphology (Supplemental Fig. S1) and that pincer‐type morphology is a feature of shape associated with moderate rHOA (Fig. 4*A*). In contrast, associations between HSMs and severe rHOA were unaffected by LCEA adjustment, consistent with our finding that the shape associated with severe rHOA showed relatively little alteration in acetabular coverage (Fig. 4*B*). Associations of higher HSM2 and lower HSM4 scores with risk of hospital‐diagnosed HOA were both partially attenuated by approximately 40% by LCEA adjustment (Table 3). LCEA adjustment also partially attenuated the association between higher HSM2 scores and risk of THR by 30% and completely attenuated the association between lower HSM4 scores and THR. These findings are consistent with the depiction of acetabular dysplasia by HSM2 and HSM4 (Supplemental Fig. S1) and our finding that acetabular dysplasia is a feature of the risk shape for both hospital‐diagnosed HOA and THR (Fig. 4 *C*, *D*).

## Discussion

Having analyzed associations between hip shape and four different definitions of HOA in more than 40,000 individuals from UK Biobank, the majority of HSMs tested showed evidence of association, which largely persisted after adjusting for age, sex, height, weight, and ethnicity. To evaluate relationships between HOA and overall hip shape and to compare shapes between definitions of HOA, we constructed composite models integrating all HSMs related to HOA outcomes. The shape associated with moderate rHOA (grade 2) comprised several features, including widening of the FN, greater acetabular coverage of the femoral head, and enlargement of the lesser and greater trochanters. However, shapes associated with OA definitions related to more severe disease differed substantially from those associated with moderate rHOA, comprising cam‐type morphology as opposed to FN widening, and reduced as opposed to increased acetabular coverage of the femoral head, suggestive of acetabular dysplasia.

Several of the SSM‐derived shape variations found to be associated with moderate rHOA have previously been reported. That said, each mode is study specific; therefore, direct comparisons with individual modes between studies cannot be made. Castano‐Betancourt and colleagues reported that a shape mode comprising a wider FN was associated with incident rHOA or THR in an SSM study of radiographs from 688 individuals.^(^ ^21^ ^)^ Our finding of greater acetabular coverage of the femoral head is in line with a previous report of a DXA SSM study by Ahedi and colleagues in which a shape mode reflecting acetabular overhang was found in prevalent rHOA.^(^ ^22^ ^)^ These findings are also consistent with our previous DXA SSM study, where a shape mode reflecting pincer‐type morphology was associated with an increased risk of prevalent rHOA.^(^ ^6^ ^)^ In previous studies, associations between greater acetabular coverage and rHOA might be explained by incorporation of acetabular osteophytes within the acetabular outline. However, this is unlikely to have been the case in the present study because acetabular osteophytes were identified and excluded before SSM, which could be done with reasonable accuracy using high‐resolution iDXA images.

Consistent with our results, the above studies also reported that enlarged lesser and greater trochanters were associated with rHOA.^(^ ^6^, ^21^, ^22^ ^)^ However, Ahedi and colleagues reported associations with what was identified as HSM2 reflecting a larger greater trochanter, and HSM6, comprising a smaller greater trochanter. Conflicting associations, when examining HSMs in isolation, justify the combination of findings from different HSMs to generate a composite model before reaching conclusions about shape relationships. That said, even when a composite model is employed, this can comprise several different aspects of altered shape, making it hard to distinguish the role of any given change. One way to overcome this is to develop subregional shape models, as exemplified by a recent study where we developed a subregional SSM of the lesser trochanter and found an association between greater size of the lesser trochanter and rHOA, consistent with the present findings.^(^ ^7^ ^)^ An alternative approach is to adjust findings for specific features measured independently, such as cam morphology and acetabular coverage, as employed in the present study.

One of the most striking differences when comparing hip shape across different definitions of HOA was a decrease in acetabular coverage, which was restricted to hospital‐diagnosed HOA and THR. That associations between HSM2 and HSM4 (which depict acetabular coverage among other features) and hospital‐diagnosed HOA and THR were partially attenuated by adjustment for LCEA supports the conclusion that reduced acetabular coverage makes an important contribution to the association between hip shape and more severe forms of HOA. Several previous studies have reported associations between acetabular coverage and HOA. For example, acetabular dysplasia, defined using LCEA, was found to be associated with increased risk of incident rHOA or THR.^(^ ^23^, ^24^, ^25^ ^)^ In previous SSM‐based studies, HSMs reflecting reduced acetabular coverage have also been associated with rHOA or THR.^(^ ^22^, ^26^ ^)^ Given that previous SSM studies found associations between reduced acetabular coverage and HOA and the fact that severe acetabular dysplasia in the form of DDH is a well‐recognized feature of early onset HOA,^(^ ^2^ ^)^ taken with our findings, acetabular dysplasia may prove a useful prognostic marker after the initial diagnosis of HOA.

A further noticeable difference between HOA severities is superior FN widening, involving the lateral aspect of the femoral head, giving a similar appearance to cam morphology. Our finding that associations between HSM3 (which depicts a cam‐type morphology among other features) and severe rHOA, HOA, and THR were partially attenuated by adjustment for AA is consistent with the suggestion that cam morphology contributed to the association between hip shape and more severe forms of HOA. Perhaps unexpectedly, the extent of this attenuation was somewhat less for THR, possibly reflecting the fact that associations with THR are estimated more imprecisely than the other outcomes because of the relatively small number of THR cases. Our findings are also consistent with those of Agricola and colleagues, who reported AA‐defined cam morphology as being associated with increased incidence of end‐stage HOA as defined by severe rHOA or THR.^(^ ^27^ ^)^ Several other studies have reported associations between cam morphology and rHOA or THR using definitions based on AA^(^ ^18^, ^24^, ^25^, ^28^ ^)^ or SSM,^(^ ^6^, ^21^, ^22^ ^)^ though without a distinction between milder and more severe forms of HOA. The present findings may reflect the fact, as for acetabular dysplasia, cam morphology predisposes to more severe forms of HOA.

Whereas our findings raise the possibility that cam morphology and acetabular dysplasia are preferentially associated with more severe forms of HOA, since those with severe HOA are likely to transition through moderate HOA, arguably one might have also expected to discern a relationship of these morphologies with risk of moderate HOA. That said, if these morphologies increase the risk of transition from moderate to severe HOA, they would be expected to account for a minority of prevalent cases of moderate HOA, leading to weaker overall relationships with moderate compared with severe HOA. Alternatively, it may be that these morphological changes are a consequence rather than a cause of HOA. This may particularly be the case with cam morphology, which might encompass modeling changes described in late‐stage HOA.^(^ ^29^ ^)^ Understanding the role of cam morphology in late‐stage OA is important because if it is not an underlying cause but a consequence, surgical correction is unlikely to be of benefit. Studies following patients with HOA as they progressively develop more severe HOA are currently lacking, making it difficult to address this question using conventional epidemiological approaches. However, using methods such as Mendelian randomization, it may be possible to examine the causal role of cam morphology in hip OA, as recently applied to study causal relationships between bone mineral density and OA.^(^ ^30^ ^)^

In terms of other distinctions, enlargement of the lesser and greater trochanters related to moderate rHOA was less marked in more severe forms of HOA. As discussed above, previous studies have generally suggested that rHOA is related to enlargement of the lesser and greater trochanters. In contrast, Agricola and colleagues reported an association between a shape mode reflecting a smaller lesser trochanter and THR,^(^ ^31^ ^)^ consistent with the present findings that suggest that a larger lesser trochanter may be a feature of milder as opposed to more severe forms of HOA. A further distinction was that in females but not males, more severe forms of HOA were related to more medial placement of the greater trochanter. We are not aware of any previous studies suggesting similar findings. Although the basis for this observation is unclear, it is conceivable that sex‐specific bone modeling responses to altered biomechanics occur in late‐stage OA, possibly related to preexisting sex differences in hip shape, which are well described.^(^ ^32^ ^)^

In interpreting shape differences associated with different definitions of HOA, we have assumed that those with clinical HOA (either hospital‐diagnosed HOA or THR) represent a progressively more severely affected subset of HOA compared with rHOA. However, in the absence of prospective data, this suggestion remains unproven. That said, prevalence decreased as expected on moving to progressively more severe OA definitions. In addition, the presence of osteophytes, which makes the major contribution to the definition of rHOA used here, is associated with an increased risk of clinical HOA features such as hip pain.^(^ ^14^ ^)^ Moreover, we found that rHOA grade ≥1 to 4 is associated with progressively stronger relationships between rHOA and hospital‐diagnosed HOA/subsequent THR.^(^ ^17^ ^)^

This study has several strengths. We investigated relationships of hip shape with different definitions of HOA in a very large study population. A further strength is that whereas associations with different HSMs can lead to conflicting conclusions about the role of different aspects of shape, our analyses were strengthened by combining the results from all associated HSMs into composite shape models. In addition, we examined the specific contribution of acetabular dysplasia and cam morphology to the observed associations between overall hip shape and HOA, by adjusting for AA and LCEA, respectively, based on an automated method for deriving these angles from points used for SSM.

A major limitation of our study is that it was based on 2D visualization of hip shape on DXA scans, hence we were only able to describe a limited proportion of overall shape variation. This may have led to spurious interpretation of shape differences, such as size of the lesser trochanter, which is affected by hip rotation. That said, limited rotation is a feature of more severe forms of HOA, whereas enlargement of the lesser trochanter was found to be more marked in less severe HOA. Another limitation is that since our analyses were based on hip DXA scans, they utilized AP views of the hip with the hip extended and adducted. This may be suboptimal for visualizing cam morphology in comparison to using lateral views or the Dunn view, where the hip is flexed and abducted. A further limitation is that models based on the acetabulum and whole proximal femur together make it difficult to determine which aspects of hip shape are relevant to disease outcomes. Future studies employing subregional models will help to further determine relevant aspects of shape related to HOA. It is also conceivable that some of the associations we observed reflected incorporation of features related to HOA into hip shape, such as a contribution of acetabular osteophytes to acetabular coverage, despite our best attempts to exclude these. However, against this suggestion, whereas greater acetabular coverage was associated with moderate HOA, acetabular coverage was reduced in more severe forms of HOA, despite the fact that osteophytes are more frequent and larger in severe compared with moderate rHOA.

In terms of other limitations, because of the observational nature of our study, it is not possible to determine whether the associations of hip shape with different definitions of HOA reflect a cause or a consequence of the disease. Certain shape characteristics that we observed, such as cam morphology, could be explained by alterations in bone modeling secondary to HOA, whereas reduced acetabular coverage is harder to attribute to this mechanism. In addition, information regarding the site of hospital‐diagnosed HOA and THR was not available, whereas hip shape was ascertained from left hip images only. However, this would have had the effect of attenuating the associations we observed rather than leading to spurious associations as a result of bias. Finally, although this study was performed in a large population‐based cohort, UK Biobank has limited ethnic diversity, and therefore our findings may not be generalizable to other populations.

In summary, we examined relationships between hip shape and HOA in ~40,000 individuals, comparing composite hip shapes according to degree of HOA severity. Moderate HOA, as reflected by rHOA grade 2, was characterized by widening of the FN, greater acetabular coverage, and enlarged lesser and greater trochanters. In more severe forms of HOA, changes reflected cam morphology, acetabular coverage was reduced, and the lesser and greater trochanters were no longer enlarged. Further analyses adjusting for AA and LCEA were consistent with the possibility that acetabular dysplasia and cam morphology contribute to the relationship between hip shape and more severe forms of HOA. We conclude that acetabular dysplasia and cam morphology are important features of severe HOA but are unrelated to less severe forms of the disease. Further studies are justified to establish the role of acetabular dysplasia and cam morphology as prognostic markers in those diagnosed with HOA.

## Disclosures

NCH has received consultancy, lecture fees, and honoraria from Alliance for Better Bone Health, Amgen, MSD, Eli Lilly, Servier, UCB, Shire, Consilient Healthcare, Kyowa Kirin, and Internis Pharma. All other authors state that they have no conflicts of interest.

## Author Contributions

**Monika Frysz:** Data curation; formal analysis; project administration; writing – original draft; writing – review and editing. **Benjamin G Faber:** Data curation; writing – original draft; writing – review and editing. **Raja Ebsim:** Data curation; methodology; writing – original draft; writing – review and editing. **Fiona R Saunders:** Data curation; writing – original draft; writing – review and editing. **Claudia Lindner:** Conceptualization; funding acquisition; methodology; writing – original draft; writing – review and editing. **Jennifer S Gregory:** Investigation; writing – original draft; writing – review and editing. **Richard M Aspden:** Funding acquisition; investigation; writing – original draft; writing – review and editing. **Nicholas C Harvey:** Funding acquisition; investigation; writing – original draft; writing – review and editing. **Tim Cootes:** Conceptualization; funding acquisition; methodology; writing – original draft; writing – review and editing. **Jon H Tobias:** Conceptualization; funding acquisition; investigation; writing – original draft; writing – review and editing.

## Ethical Approval Statement

This study was approved by UK Biobank, which is overseen by the Ethics Advisory Committee and received approval from the National Information Governance Board for Health and Social Care and Northwest Multi‐Centre Research Ethics Committee (11/NW/0382). All participants provided informed consent for this study.

### Peer Review

The peer review history for this article is available at https://publons.com/publon/10.1002/jbmr.4649.

## Supporting information


**Appendix S1.** Supplemental Information

## Data Availability

The data from this study will be available from UK Biobank in a forthcoming data release. Users must be registered with UK Biobank to access their resources (https://bbams.ndph.ox.ac.uk/ams/).
