# Supplementary material for: Machine Learning–Derived Acetabular Dysplasia and Cam Morphology Are Features of Severe Hip Osteoarthritis: Findings From UK Biobank
Source: J Bone Miner Res. 2022 Aug 7;37(9):1720–32. doi: 10.1002/jbmr.4649 (PMC9545366; doi:10.1002/jbmr.4649)
Supplement: Supplementary file 1 — Appendix S1. Supplemental Information [file JBMR-37-1720-s001.docx]

## Supplementary Tables and Figures

Supplementary Table 1: Variation explained by the first 10 hip shape modes.

| **Hip Shape Mode** | **% Variance** | **Cumulative variance** |
| --- | --- | --- |
| 1 | 26.6 | 26.6 |
| 2 | 21.5 | 48.1 |
| 3 | 10.6 | 58.7 |
| 4 | 7.5 | 66.2 |
| 5 | 6.5 | 72.6 |
| 6 | 4.6 | 77.2 |
| 7 | 2.9 | 80.0 |
| 8 | 2.3 | 82.4 |
| 9 | 2.1 | 84.5 |
| 10 | 1.8 | 86.3 |


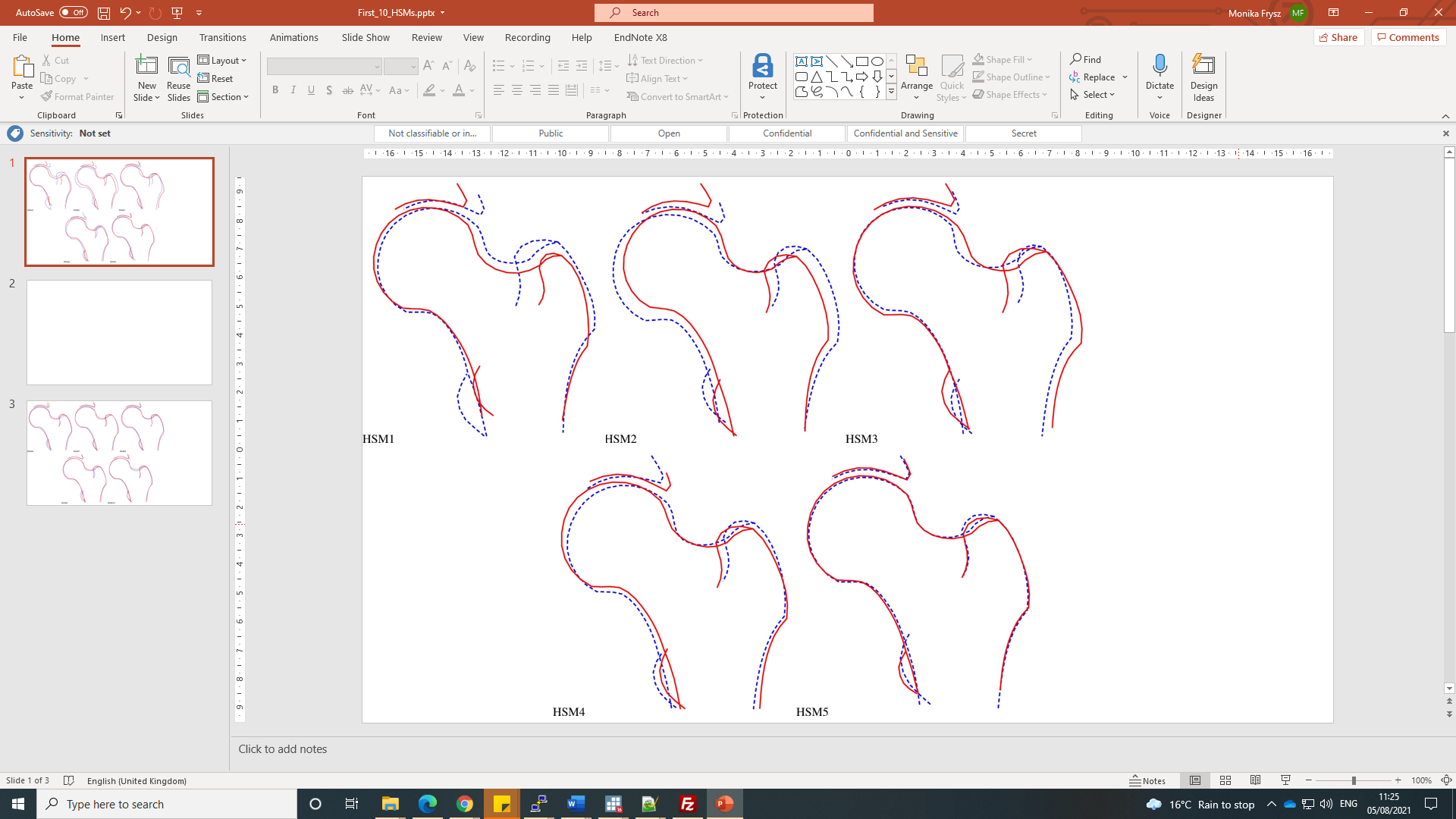


Supplementary Figure 1: Variation in hip shape described by modes 1 - 5, red line shows +2 SDs blue dashed line -2 SDs.


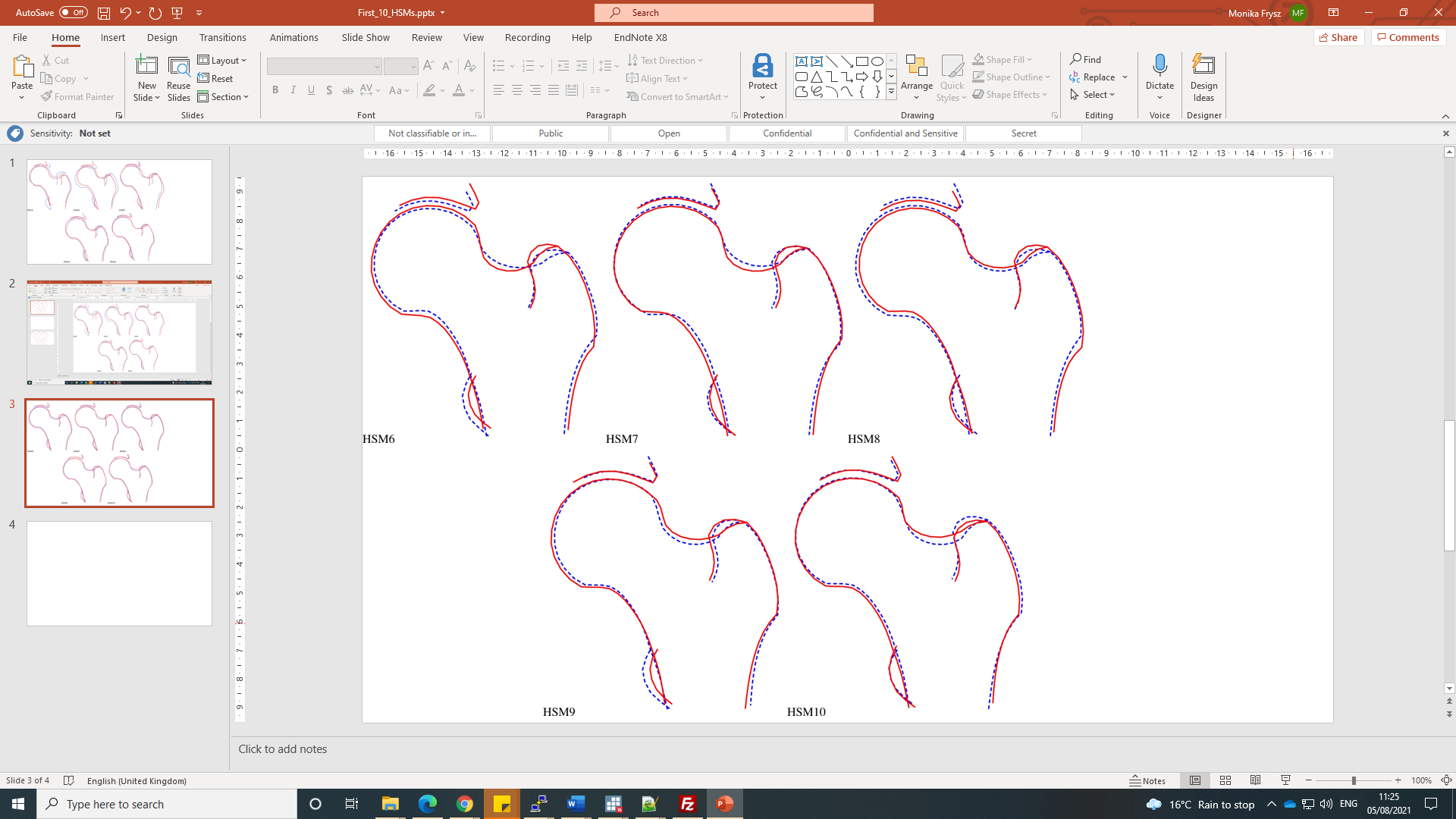


Supplementary Figure 2: Variation in hip shape described by modes 6 -10, red line shows +2 SDs blue dashed line -2 SDs.

Supplementary Figure 3


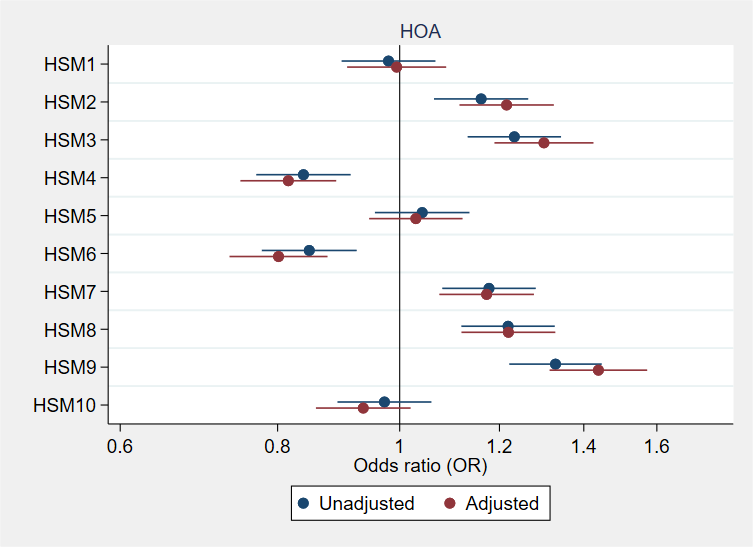

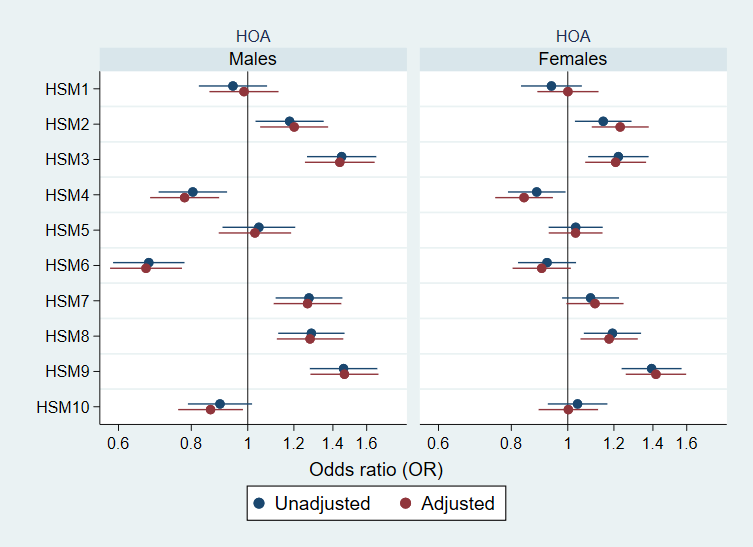


B

A

Associations between hip shape and hospital diagnosed hip OA; combined (A) and stratified by sex (B). Results are odds ratios (ORs) of outcome per SD increase in hip shape mode (HSM) score and 95% confidence interval (CI). Model adjusted for age, sex, height, weight, and ethnicity (categorised into binary variable white /other).

Supplementary Figure 4


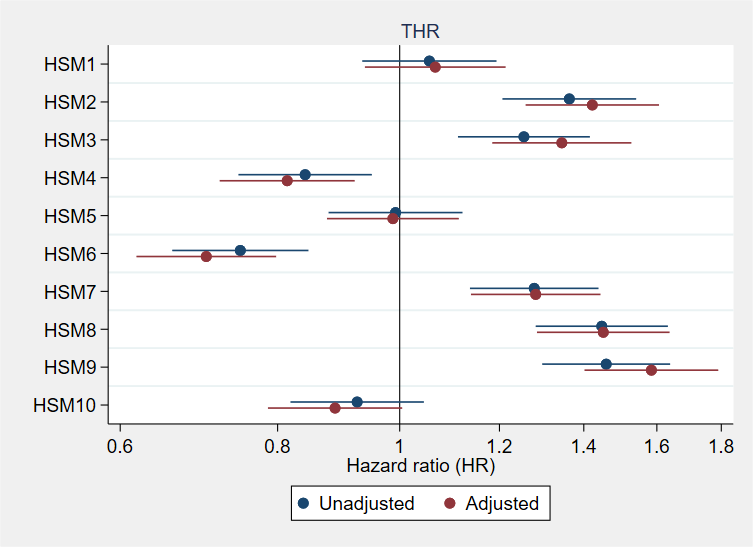

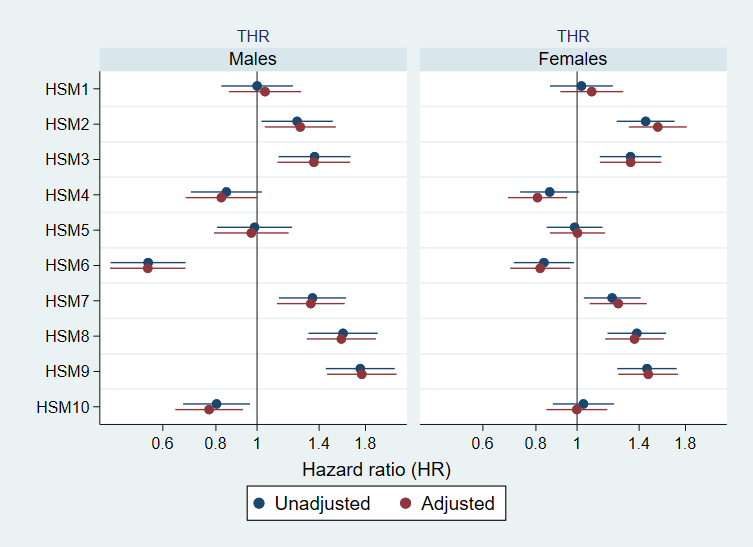


B

A

Associations between hip shape and THR; combined (A) and stratified by sex (B). THR: Total hip replacement. Results are Hazard ratios (HRs) of outcome per SD increase in hip shape mode (HSM) score and 95% confidence interval (CI). Model adjusted for age, sex, height, weight, and ethnicity (categorised into binary variable white /other).
